# Low Time-Weighted Parathyroid Hormone Is Associated with Malnutrition-Inflammation and Functional Dependence in Maintenance Hemodialysis

**DOI:** 10.3390/nu18162742

**Published:** 2026-08-21

**Authors:** Nomy Levin Iaina, Alexandra Petkutsa, Nayaf Habashi, Evgenia Gurevich

**Affiliations:** 1 Department of Nephrology and Hypertension, Barzilai University Medical Center, Ashkelon 7830604, Israel; 2 School of Medicine, Faculty of Health Sciences, Ben Gurion University of the Negev, Be’er Sheva 8410501, Israel; jenyg@bmc.gov.il; 3 Department of Anesthesiology, Barzilai University Medical Center, Ashkelon 7830604, Israel; alexandrape@bmc.gov.il; 4 Department of Nephrology, HaEmeq Hospital Afula, Afula 1834111, Israel; nayef_ha@clalit.org.il; 5 Clalit Health Services, Israel; 6 Rappaport Faculty of Medicine, Technion-Israel Institute of Technology, Haifa 3200003, Israel; 7 Pediatrics Department, Barzilai University Medical Center, Ashkelon 7830604, Israel

**Keywords:** parathyroid hormone, time-weighted PTH, hemodialysis, malnutrition-inflammation, functional status, mortality

## Abstract

*Background/Objectives*: Low parathyroid hormone (PTH) levels in maintenance hemodialysis may reflect malnutrition, inflammation, reduced functional reserve, or low bone turnover rather than optimal CKD-MBD control. We evaluated associations of longitudinal PTH burden with nutritional-inflammatory and functional-dependence profiles, hospitalization, and mortality. *Methods*: This retrospective cohort included 188 adults receiving maintenance hemodialysis. Serial PTH measurements were summarized using time-weighted mean PTH. Associations with nutritional-inflammatory biomarkers, functional-dependence measures, and hospitalization were assessed using regression models. Mortality was primarily evaluated using a joint longitudinal–survival model linking repeated log2-transformed PTH measurements with time to death and adjusting for age, sex, dialysis vintage, and dialysis access. A 6-month landmark analysis was performed as a complementary sensitivity analysis. *Results*: Median time-weighted mean PTH was 292 pg/mL. Lower longitudinal PTH burden was associated with lower albumin, cholesterol, and transferrin and higher *C*-reactive protein and ferritin. Higher PTH was associated with lower odds of worse mobility and dependence in dressing, bathing, toileting, and continence after adjustment. Hospitalization was not significantly associated with PTH. During follow-up, 62 patients died. In the joint model, each twofold higher estimated current PTH level was associated with a lower observed mortality hazard (adjusted hazard ratio [HR] 0.58, 95% confidence interval [CI] 0.46–0.73; *p* < 0.001). The 6-month landmark analysis included 159 patients and 44 subsequent deaths. Each twofold higher PTH level was associated with lower subsequent mortality hazard (HR 0.69, 95% CI 0.51–0.95; *p* = 0.021), while PTH < 150 pg/mL was associated with higher mortality than PTH 300–600 pg/mL (HR 2.84, 95% CI 1.22–6.63). *Conclusions*: Low time-weighted PTH was associated with adverse nutritional-inflammatory and functional profiles, and lower estimated current PTH with higher mortality. Findings were consistent in the 6-month landmark analysis, do not establish causality, and support interpreting PTH in the broader clinical context.

## 1. Introduction

Chronic kidney disease-mineral and bone disorder (CKD-MBD) is a systemic complication of advanced chronic kidney disease and kidney failure, characterized by disturbances in calcium, phosphate, parathyroid hormone (PTH), vitamin D, and fibroblast growth factor-23 metabolism, together with abnormalities in bone turnover, mineralization, vascular calcification, and cardiovascular risk [1,2,3].

In patients receiving maintenance hemodialysis, secondary hyperparathyroidism is one of the most clinically relevant manifestations of CKD-MBD. Although PTH is widely used to guide treatment, contemporary guidelines emphasize interpretation of serial trends and integrated assessment of calcium, phosphate, PTH, bone disease, and cardiovascular risk rather than reliance on a single biochemical value [1,4].

Elevated PTH has traditionally been viewed as a marker of high-turnover bone disease and has been associated with mortality, fractures, vascular calcification, and cardiovascular morbidity in dialysis populations [3,4,5]. However, the relationship between PTH and outcomes is complex and may be non-linear. Observational data suggest that both very high and relatively low PTH levels are associated with adverse outcomes, while low PTH may reflect malnutrition-inflammation, comorbidity burden, or low bone turnover rather than favorable CKD-MBD control [5,6].

In this single-center retrospective cohort study, we aimed to evaluate whether longitudinal PTH burden, assessed using repeated PTH measurements and summarized primarily by time-weighted mean PTH, was associated with nutritional-inflammatory biomarkers and routinely documented functional-dependence measures in patients receiving maintenance hemodialysis. We additionally examined its associations with hospitalization burden and all-cause mortality using analytical approaches designed for longitudinal biomarker data.

## 2. Materials and Methods

### 2.1. Study Design and Setting

We conducted a retrospective single-center cohort study among adult patients receiving maintenance hemodialysis at the dialysis unit of the Department of Nephrology and Hypertension, Barzilai University Medical Center, Ashkelon, Israel, between 1 January 2022 and 31 December 2024. This study was designed to evaluate associations between longitudinal PTH burden and nutritional-inflammatory and functional-dependence measures, as well as clinically relevant outcomes.

Nutritional-inflammatory biomarkers and routinely documented measures of mobility and activities of daily living were evaluated as related clinical outcomes. All-cause mortality was the prespecified primary clinical outcome, whereas hospitalization burden was evaluated as an exploratory outcome. This manuscript was prepared in accordance with the STROBE reporting guideline for observational cohort studies.

### 2.2. Study Population

Eligible patients were adults aged 18 years or older who had received maintenance hemodialysis for at least 3 months. For patients transferred from other dialysis centers, eligibility was based on the total duration of maintenance hemodialysis, including treatment received before transfer to our unit. The dialysis start date recorded in this study database reflected initiation of hemodialysis at our center.

Patients receiving acute or temporary hemodialysis, including those who initiated dialysis during an acute hospitalization or required kidney replacement therapy for acute kidney injury, were excluded, as were patients with less than three months of maintenance hemodialysis treatment. Hospitalizations occurring after cohort entry were fully captured and included in the follow-up analyses.

The analytic cohort included patients with available PTH measurements and relevant clinical, laboratory, functional, hospitalization, and survival data. Study entry was defined as 1 January 2022 for prevalent patients or the first date of eligibility during the study period for patients who entered later. Patients were followed from study entry until death, dialysis discontinuation or transfer, or 31 December 2024, whichever occurred first. For survival analyses, time origin was defined according to the specific analytical approach, as described below.

### 2.3. PTH Exposure Definition

Because repeated PTH measurements were accumulated during follow-up, different analytical approaches were used according to the outcome of interest. Time-weighted mean PTH served as the principal summary measure of longitudinal PTH burden for analyses of nutritional-inflammatory biomarkers, functional outcomes, and hospitalization. Mortality was evaluated primarily using a joint longitudinal-survival model based on repeated PTH measurements, as described in the Statistical Analysis Section, whereas time-weighted mean PTH remained the primary longitudinal summary measure for analyses of nutritional-inflammatory biomarkers, functional outcomes, and hospitalization. Conventional Cox analyses using time-weighted mean PTH were performed as complementary descriptive analyses to facilitate comparison with previous studies.

Time-weighted mean PTH was calculated from serial PTH measurements obtained during follow-up using linear interpolation between consecutive measurements; the area under the PTH-time curve was divided by the duration of the PTH observation window. Time-weighted mean PTH was selected a priori as the primary longitudinal summary because it assigns weight according to the duration of the intervals between consecutive measurements. It therefore accounts explicitly for unequal measurement spacing and reduces the disproportionate influence of clusters of closely spaced measurements.

In contrast, the arithmetic mean assigns equal weight to each available measurement, irrespective of the duration represented by that measurement. This methodological rationale was not intended to imply that time-weighted averaging is clinically superior to arithmetic averaging. For patients with only one PTH measurement, that value was assigned as the time-weighted mean PTH. The patient-specific PTH ascertainment window extended from the first to the last available PTH measurement during follow-up and therefore varied according to survival duration and data availability. For patients with only one PTH measurement, the PTH observation window was defined as zero days.

Time-weighted mean PTH was analyzed continuously, per 100 pg/mL higher PTH, and according to clinical categories: <150, 150–300, 300–600, and >600 pg/mL. For survival analyses, it was also analyzed according to quartiles. Arithmetic mean PTH was calculated for comparison with time-weighted mean PTH. Arithmetic mean PTH was retained as a simpler comparator and was used in sensitivity analyses to assess whether the clinical findings depended on the method used to summarize serial PTH measurements.

Additional longitudinal PTH measures were calculated for descriptive and exploratory analyses, including arithmetic mean PTH, PTH standard deviation, coefficient of variation, percentage of time within the PTH range of 150–600 pg/mL, and percentage of time above 600 pg/mL. Time in range and time above 600 pg/mL were estimated using linear interpolation between consecutive measurements.

### 2.4. Data Collection and Outcomes

Data were extracted retrospectively from dialysis electronic medical records and included demographic characteristics, dialysis-related parameters, laboratory data, CKD-MBD treatments, nutritional and inflammatory markers, functional assessments, hospitalization counts, and mortality status. CKD-MBD treatments included phosphate binders, vitamin D derivatives, cinacalcet, etelcalcetide, and calcium therapy. Dialysis-related variables included dialysis vintage, vascular access type, number and duration of dialysis sessions, weekly dialysis time, dry weight, and Kt/V. Laboratory variables included markers of mineral metabolism, nutritional status, inflammation, anemia, and lipid profile.

### 2.5. Nutritional and Inflammatory Assessment

Nutritional-inflammatory status was assessed using serum albumin, phosphorus, total cholesterol, triglycerides, transferrin, ferritin, *C*-reactive protein (CRP), hemoglobin, calcium, and iron. Clinical nutritional assessment included Subjective Global Assessment (SGA) and Malnutrition Universal Screening Tool (MUST) scores when available. These variables were used to characterize the nutritional-inflammatory profile associated with longitudinal PTH burden. SGA and MUST scores were analyzed as exploratory continuous outcomes in relation to mean PTH.

Continuous associations between time-weighted mean PTH and nutritional-inflammatory biomarkers were assessed using linear regression, and biomarker distributions were compared across time-weighted mean PTH categories. Because time-weighted mean PTH was derived from measurements accumulated over the follow-up period and could therefore include PTH values obtained after the corresponding biochemical assessment, these analyses were intended as descriptive association analyses between a longitudinal PTH summary and routinely collected biochemical measures. They should not be interpreted as cross-sectional or temporally ordered exposure-outcome analyses, and no directionality or causality can be inferred from these associations. In addition, because direct measures of dietary intake, body composition, muscle mass, and muscle strength were not routinely available, the nutritional-inflammatory pattern was defined using routinely collected biochemical and clinical indicators rather than formal diagnostic criteria for protein-energy wasting or sarcopenia.

### 2.6. Functional Status

Functional status was evaluated as a key patient-centered outcome. Functional variables were obtained from routine clinical assessments recorded in the dialysis electronic medical record as part of standard care; for each patient, the assessment closest to study entry was used. These assessments included gait stability, mobility aid use, overall mobility level, history of falls, dressing, bathing, toileting, continence status, and Norton score. Norton score was analyzed as an exploratory continuous outcome, with lower scores indicating greater risk of pressure ulcer development. Similarly, the functional analyses were designed to evaluate associations between routinely documented functional status and longitudinal PTH burden rather than to assess the effect of preceding PTH levels on subsequent functional outcomes.

Gait instability, mobility aid use, assistance with dressing, assistance with bathing, and fall history were analyzed as binary outcomes. Overall mobility, toileting function, and continence status were analyzed as ordinal outcomes, with higher categories indicating worse function. Coding direction was harmonized so that higher outcome categories consistently represented greater impairment.

These measures were considered pragmatic indicators of functional dependence relevant to nutritional vulnerability and loss of independence in maintenance hemodialysis. These variables were derived from routine clinical documentation and were not components of a validated composite frailty scale, a formal sarcopenia assessment, an objective physical-performance battery, or a patient-reported quality-of-life instrument. They were therefore interpreted as individual pragmatic indicators of functional dependence rather than as diagnostic measures of frailty, sarcopenia, or health-related quality of life. Missingness of routinely documented functional assessments was additionally evaluated across clinical time-weighted mean PTH categories. The proportion of missing observations for each functional measure was compared across categories using the Fisher–Freeman–Halton exact test.

### 2.7. Clinical Outcomes

The prespecified primary clinical outcome was all-cause mortality. The primary mortality analysis used a joint longitudinal-survival model based on repeated PTH measurements, whereas conventional Cox analyses using time-weighted mean PTH were performed as complementary descriptive analyses.

### 2.8. Statistical Analysis

Continuous variables are presented as mean ± standard deviation (SD) or median with interquartile range (IQR), according to their distribution. Categorical variables are presented as counts and percentages. Normality was assessed using the Shapiro–Wilk test together with visual inspection of histograms and Q-Q plots. Between-group comparisons were performed using one-way analysis of variance (ANOVA) or the Kruskal–Wallis test for continuous variables and the chi-square or Fisher’s exact test for categorical variables, as appropriate.

For analyses across clinical PTH categories, patients were classified according to time-weighted mean PTH as <150, 150–300, 300–600, and >600 pg/mL, reflecting clinically relevant CKD-MBD treatment thresholds. Quartiles of time-weighted mean PTH were additionally evaluated in mortality analyses. To reduce the probability of false-positive findings arising from multiple comparisons, false discovery rate (FDR) correction using the Benjamini–Hochberg procedure was applied separately to the nutritional-inflammatory biomarker analyses and to the functional-dependence analyses.

Associations between time-weighted mean PTH and continuous nutritional-inflammatory biomarkers were evaluated using univariable linear regression. Because CRP and ferritin demonstrated right-skewed distributions, continuous regression analyses for these biomarkers were performed using log-transformed values (log1p for CRP and natural logarithm for ferritin). Binary functional outcomes, including mobility, falls, and dependence in activities of daily living, were analyzed using multivariable logistic regression. Hospitalization burden was evaluated as the number of hospitalizations per patient during follow-up.

Correlations between PTH and hospitalization burden were assessed using Pearson and Spearman correlation coefficients. Hospitalization rates were additionally analyzed using Poisson regression with follow-up duration included as an offset. Adjusted linear, logistic, and Poisson models included age, sex, dialysis vintage, and dialysis access.

Because PTH was measured repeatedly during follow-up, the primary mortality analysis was performed using a joint longitudinal-survival model to account for the endogenous longitudinal evolution of PTH and the informative termination of repeated PTH measurements by death. The longitudinal sub model included all available serial intact PTH measurements, modeled on the log2 scale using a linear mixed-effects model with patient-specific random intercepts and random slopes. The survival sub model evaluated time from the first available PTH measurement to all-cause mortality and included age, sex, dialysis vintage, and dialysis access as baseline covariates.

The longitudinal and survival sub models were linked through the estimated current underlying PTH value. Hazard ratios are presented per twofold higher estimated current PTH level. Accordingly, the association parameter from the joint model reflects the relationship between the estimated current underlying PTH level and mortality hazard and should not be interpreted as an association with whole-follow-up time-weighted mean or cumulative PTH burden.

A post hoc 6-month landmark analysis was performed as a complementary sensitivity analysis to establish temporal precedence between PTH ascertainment and mortality follow-up. Time-weighted mean PTH was calculated using only measurements obtained during the first six months after the initial PTH measurement. Patients who died or were censored before the landmark were excluded, and mortality follow-up began at the six-month landmark.

Cox proportional hazards models were fitted unadjusted and adjusted for age, sex, dialysis vintage, and dialysis access. Additional sensitivity analyses were performed after restricting the landmark cohort to patients with at least two PTH measurements during the six-month ascertainment window. Covariates for the adjusted models were selected a priori based on clinical knowledge and biological plausibility and the need to avoid over-adjustment.

Variables that could plausibly represent downstream consequences of longitudinal PTH status, treatment response, or the patient’s evolving clinical condition—including nutritional-inflammatory biomarkers and CKD-MBD therapies—were not included in the primary adjustment models in order to avoid potential over-adjustment.

To facilitate comparison with previous studies and to illustrate the observed longitudinal associations, conventional time-to-event analyses based on time-weighted mean PTH were also performed as secondary descriptive analyses. Kaplan–Meier curves were compared using the log-rank test. Cox proportional hazards regression was used to estimate hazard ratios for continuous PTH, quartiles, and clinically defined PTH categories, adjusted for age, sex, dialysis vintage, and dialysis access. Restricted cubic spline regression with four knots was used to explore potential non-linear associations between time-weighted mean PTH and mortality. Because time-weighted mean PTH was calculated from measurements accumulated during follow-up, these analyses were interpreted as descriptive longitudinal association analyses rather than baseline prognostic models.

To evaluate whether the method used to summarize longitudinal PTH influenced the findings, all mortality and hospitalization analyses were repeated using the arithmetic mean of serial PTH measurements. The relationship between arithmetic mean PTH and time-weighted mean PTH was assessed using Pearson and Spearman correlation coefficients, whereas categorical concordance and Bland–Altman analysis were used to evaluate agreement.

Missing laboratory values were not imputed. Analyses were performed using available-case data. Patients contributed all available PTH measurements to the longitudinal analyses. Because functional assessments were extracted from routine clinical documentation rather than collected prospectively according to a study protocol, missing values were assumed to reflect the absence of documented assessments rather than planned missingness. Statistical significance was defined as a two-sided *p*-value < 0.05. Statistical analyses were performed using Python version 3.13.5.

### 2.9. Ethical Considerations

This study was approved by the institutional Helsinki committee of Barzilai University Medical Center. Given the retrospective design and use of coded clinical data, the requirement for informed consent was waived. All data were de-identified before analysis and handled according to institutional privacy regulations.

## 3. Results

### 3.1. Study Cohort and Distribution of PTH

This study included 188 patients receiving maintenance hemodialysis between 1 January 2022 and 31 December 2024. Patients were followed until death, dialysis discontinuation or transfer, or the end of the study period. Patient demographic, dialysis-related, biochemical, and CKD-MBD treatment characteristics according to time-weighted mean PTH categories are presented in Table 1.

**Figure 1:**
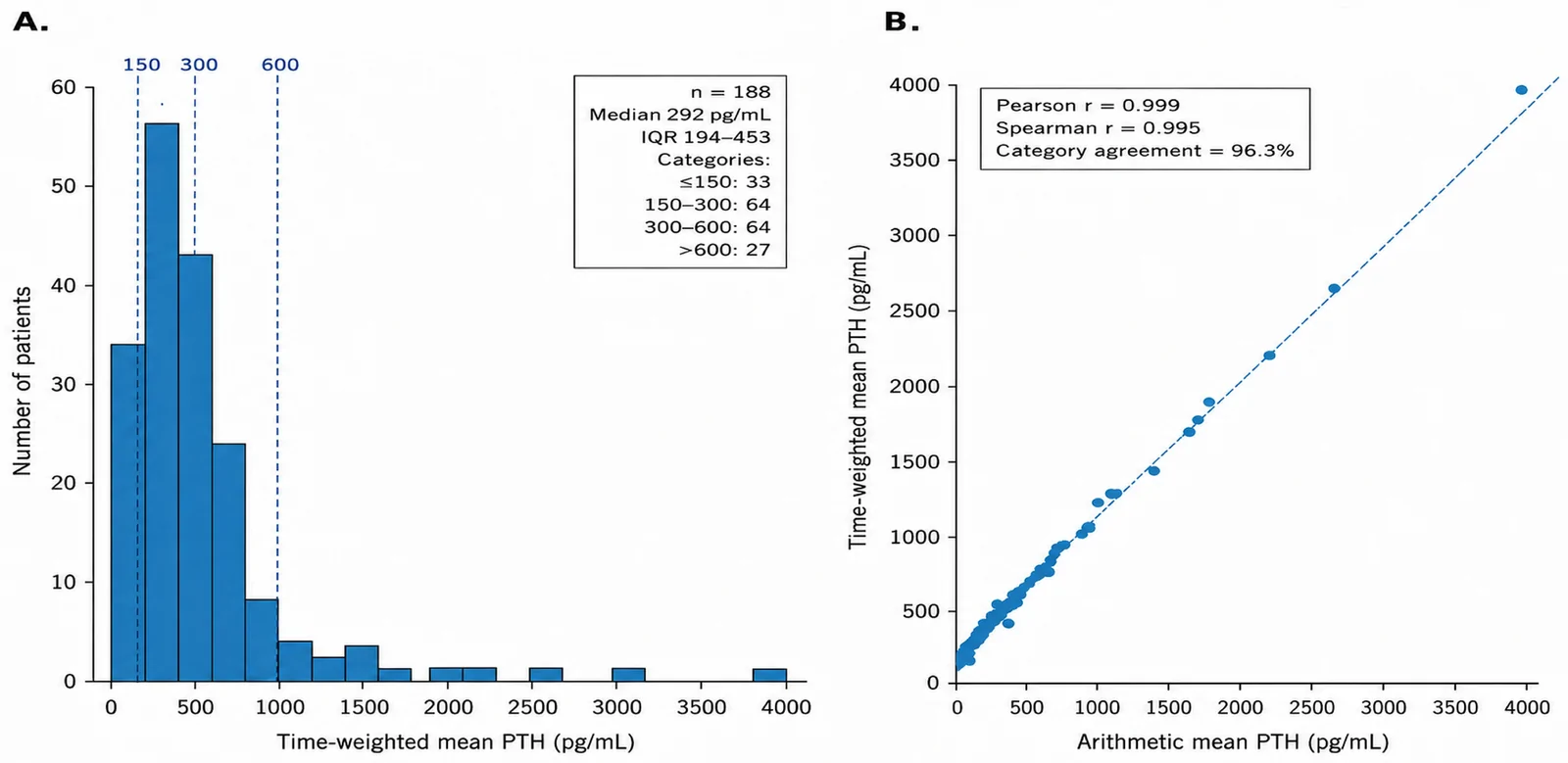
Distribution of longitudinal PTH burden and comparison of arithmetic and time-weighted mean PTH. (**A**) Distribution of time-weighted mean parathyroid hormone (PTH) among 188 patients receiving maintenance hemodialysis. Time-weighted mean PTH was calculated from serial PTH measurements using linear interpolation between consecutive measurements. Dashed vertical lines indicate the prespecified clinical thresholds of 150, 300, and 600 pg/mL. Median time-weighted mean PTH was 292 pg/mL (IQR 194–453). (**B**) Relationship between arithmetic mean PTH and time-weighted mean PTH. The dashed diagonal line represents perfect equality. The two measures were highly correlated (Pearson r = 0.999; Spearman ρ = 0.995), and 181 of 188 patients (96.3%) were assigned to the same clinical PTH category using both methods. Absolute agreement was evaluated separately using Bland–Altman analysis (Supplementary Figure S1).

Patients contributed a median of 7 PTH measurements during follow-up (IQR 4–13; range 1–24). Time-weighted mean PTH was widely distributed, with a median of 292 pg/mL (IQR 194–453; range 9–3971 pg/mL). According to the prespecified clinical categories, 33 patients (17.6%) had time-weighted mean PTH < 150 pg/mL, 64 (34.0%) had PTH 150–300 pg/mL, 64 (34.0%) had PTH 300–600 pg/mL, and 27 (14.4%) had PTH > 600 pg/mL (Table 1 and Figure 1A).

Arithmetic mean PTH had a similar distribution, with a median of 292 pg/mL (IQR 202–459). Arithmetic and time-weighted mean PTH were highly correlated (Pearson r = 0.999; Spearman ρ = 0.995), and 181 of 188 patients (96.3%) were assigned to the same clinical PTH category using both methods (Figure 1B). In the Bland–Altman analysis, the mean difference between time-weighted and arithmetic mean PTH was −0.11 pg/mL, with 95% limits of agreement from −51.15 to 50.93 pg/mL (Supplementary Figure S1). Despite the near-perfect correlation between the two summary measures, the Bland–Altman analysis demonstrated minimal average bias, although some individual-level variation remained. Additional descriptive longitudinal PTH measures, including variability and time within the prespecified PTH ranges, are presented in Table 1.

### 3.2. Association of Time-Weighted Mean PTH with Nutritional and Inflammatory Biomarkers

Lower longitudinal PTH burden was consistently associated with a less favorable nutritional-inflammatory biomarker profile (Table 2 and Table 3). In unadjusted linear regression analyses, each 100 pg/mL higher time-weighted mean PTH was significantly associated with higher serum albumin, phosphorus, transferrin, and total cholesterol. No significant continuous associations were observed with hemoglobin, calcium, triglycerides, iron, or *C*-reactive protein (CRP). Log-transformed ferritin showed a modest inverse association with time-weighted mean PTH (β = −0.033 per 100 pg/mL higher PTH, 95% CI −0.066 to −0.0004; *p* = 0.048), although this association did not remain statistically significant after FDR correction (q = 0.102). Log1p-transformed CRP was not significantly associated with time-weighted mean PTH (Table 3).

Analyses according to clinical PTH categories demonstrated significant between-group differences in nutritional and inflammatory biomarkers. Patients in the lower PTH categories generally had lower albumin, cholesterol, and transferrin concentrations and higher inflammatory-marker levels. Median CRP was 25.0 mg/L in the <150 pg/mL group and 9.5 mg/L in the >600 pg/mL group, while median ferritin was 507 and 351 ng/mL, respectively. Serum phosphorus also differed significantly across PTH categories, ranging from a median of 5.1 mg/dL in the <150 pg/mL group to 6.8 mg/dL in the >600 pg/mL group (Table 2). After Benjamini–Hochberg false discovery rate correction, the associations of time-weighted mean PTH with albumin, phosphorus, and transferrin remained statistically significant, whereas the association with total cholesterol did not. All comparisons across clinical PTH categories remained statistically significant after correction (Table 2 and Table 3).

Clinical nutritional assessment scores were available only in a subset of patients. In exploratory analyses, time-weighted mean PTH was not significantly associated with either the Malnutrition Universal Screening Tool (MUST) score (β = 0.009 per 100 pg/mL, 95% CI −0.043 to 0.060; *p* = 0.741) or the Subjective Global Assessment (SGA) score (β = 0.010, 95% CI −0.045 to 0.065; *p* = 0.715).

Overall, these findings indicate that lower longitudinal PTH burden clustered with biochemical features consistent with reduced nutritional reserve and greater inflammatory burden.

### 3.3. Association of Time-Weighted Mean PTH with Mobility and Daily Functional Status

**Figure 2:**
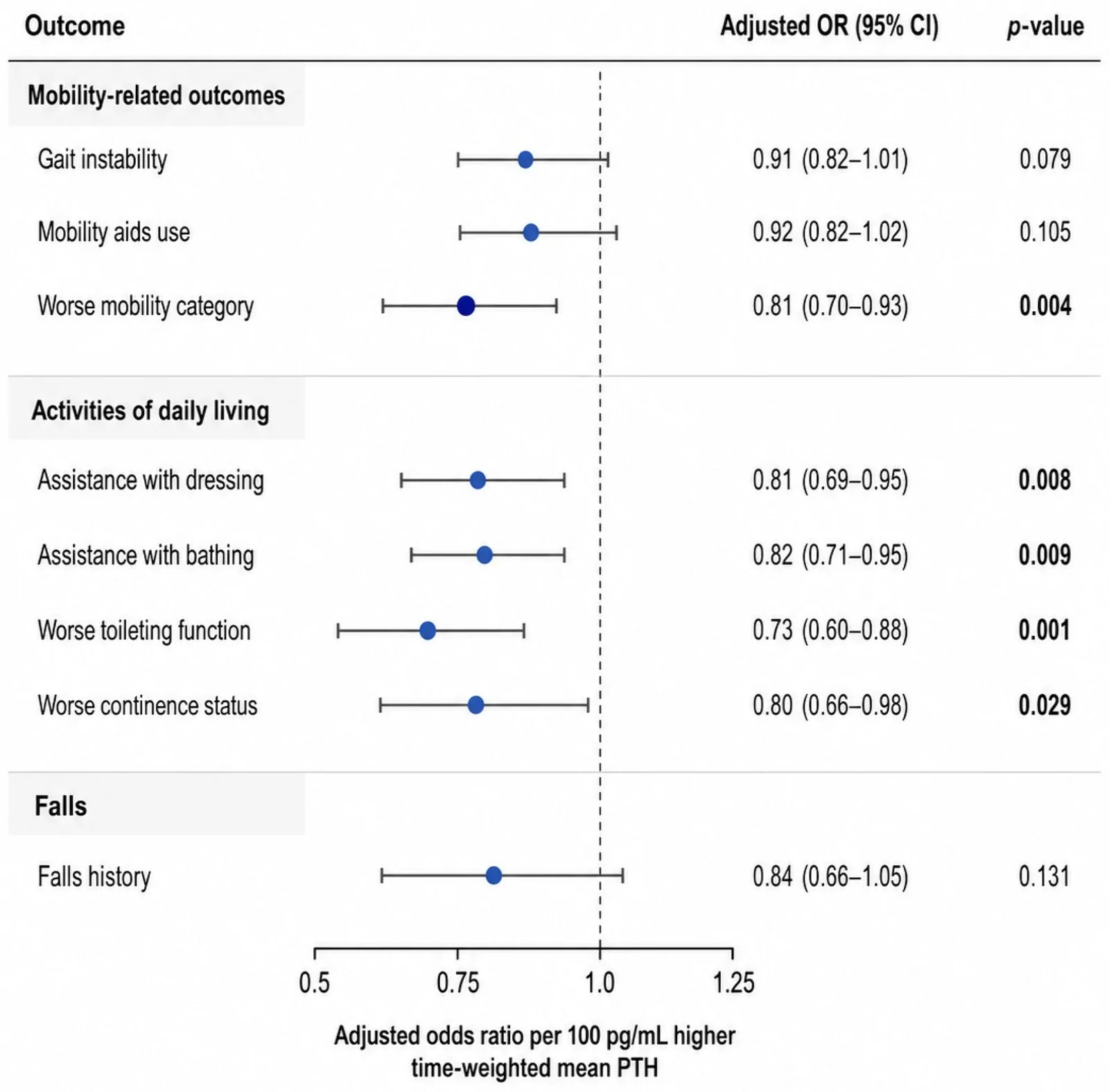
Adjusted associations between time-weighted mean PTH and mobility and functional-dependence outcomes. Forest plot showing adjusted odds ratios (ORs) per 100 pg/mL higher time-weighted mean PTH for routinely documented mobility and activities-of-daily-living outcomes. Models were adjusted for age, sex, dialysis vintage, and dialysis access. Odds ratios below 1 indicate lower odds of worse functional status with higher longitudinal PTH burden. Error bars represent 95% confidence intervals.

Lower longitudinal PTH burden was associated with greater functional impairment and dependence in activities of daily living (Table 4 and Figure 2). In unadjusted analyses, higher time-weighted mean PTH was associated with lower odds of gait instability, mobility aid use, worse mobility category, assistance with dressing or bathing, worse toileting function, and worse continence status.

Missingness of functional assessments differed across time-weighted mean PTH categories and was generally more frequent in the lower PTH groups (Supplementary Table S2). Outcome-specific missingness showed this pattern for several functional measures.

After adjustment for age, sex, dialysis vintage, and dialysis access, higher time-weighted mean PTH remained independently associated with lower odds of worse mobility category (adjusted OR 0.81 per 100 pg/mL, 95% CI 0.70–0.93; *p* = 0.004), assistance with dressing (OR 0.81, 95% CI 0.69–0.95; *p* = 0.008), assistance with bathing (OR 0.82, 95% CI 0.71–0.95; *p* = 0.009), worse toileting function (OR 0.73, 95% CI 0.60–0.88; *p* = 0.001), and worse continence status (OR 0.80, 95% CI 0.66–0.98; *p* = 0.029). Associations with gait instability, mobility aid use, and history of falls were attenuated after adjustment and were no longer statistically significant. Following Benjamini–Hochberg false discovery rate correction, the adjusted associations with worse mobility category, dressing, bathing, toileting, and continence remained statistically significant, whereas gait instability, mobility aid use, and falls did not (Table 4).

Norton score was analyzed as an exploratory continuous outcome. Higher time-weighted mean PTH was associated with higher Norton scores, indicating better overall functional status and lower pressure-ulcer risk (β = 0.18 per 100 pg/mL, 95% CI 0.07–0.29; *p* = 0.002).

Because lower Norton scores indicate greater risk of pressure ulcer development, this finding was consistent with poorer overall functional and clinical status among patients with lower PTH levels. The association remained statistically significant after adjustment for age, sex, dialysis vintage, and vascular access.

Overall, lower longitudinal PTH burden was consistently associated with greater documented functional dependence across multiple routine clinical assessments, supporting the association between low PTH and reduced functional reserve in maintenance hemodialysis patients. All five adjusted functional associations remained statistically significant after false discovery rate correction (Table 4).

### 3.4. Association of Time-Weighted Mean PTH with Hospitalization Burden

Hospitalization burden was evaluated as an exploratory clinical outcome. The median number of hospitalizations during follow-up was 4 (IQR 2–8.25; range 0–22). Hospitalization counts were not significantly associated with time-weighted mean PTH (Pearson r = −0.050, *p* = 0.494; Spearman rho = −0.060, *p* = 0.414). Because follow-up duration differed between patients, hospitalization rates were additionally evaluated using Poisson regression with follow-up time included as an offset.

In the unadjusted analysis, each 100 pg/mL higher time-weighted mean PTH was associated with a non-significant trend toward lower hospitalization rates. This association remained non-significant after adjustment for age, sex, dialysis vintage, and dialysis access (adjusted incidence rate ratio [IRR] 0.97, 95% CI 0.94–1.00; *p* = 0.086). Overall, longitudinal PTH burden was not significantly associated with hospitalization burden in this cohort.

### 3.5. Mortality Analyses

Mortality was evaluated using three complementary analytical approaches. The primary analysis used a joint longitudinal-survival model, followed by a 6-month landmark sensitivity analysis and secondary descriptive Cox analyses based on time-weighted mean PTH.

#### 3.5.1. Primary Mortality Analysis (Joint Longitudinal-Survival Model)

During follow-up, 62 of 188 patients (33.0%) died. The primary mortality analysis was performed using a joint longitudinal-survival model linking repeated PTH measurements with time to death. The longitudinal component incorporated all available serial PTH measurements modeled on the log2 scale, whereas the survival component was adjusted for age, sex, dialysis vintage, and dialysis access.

**Figure 3:**
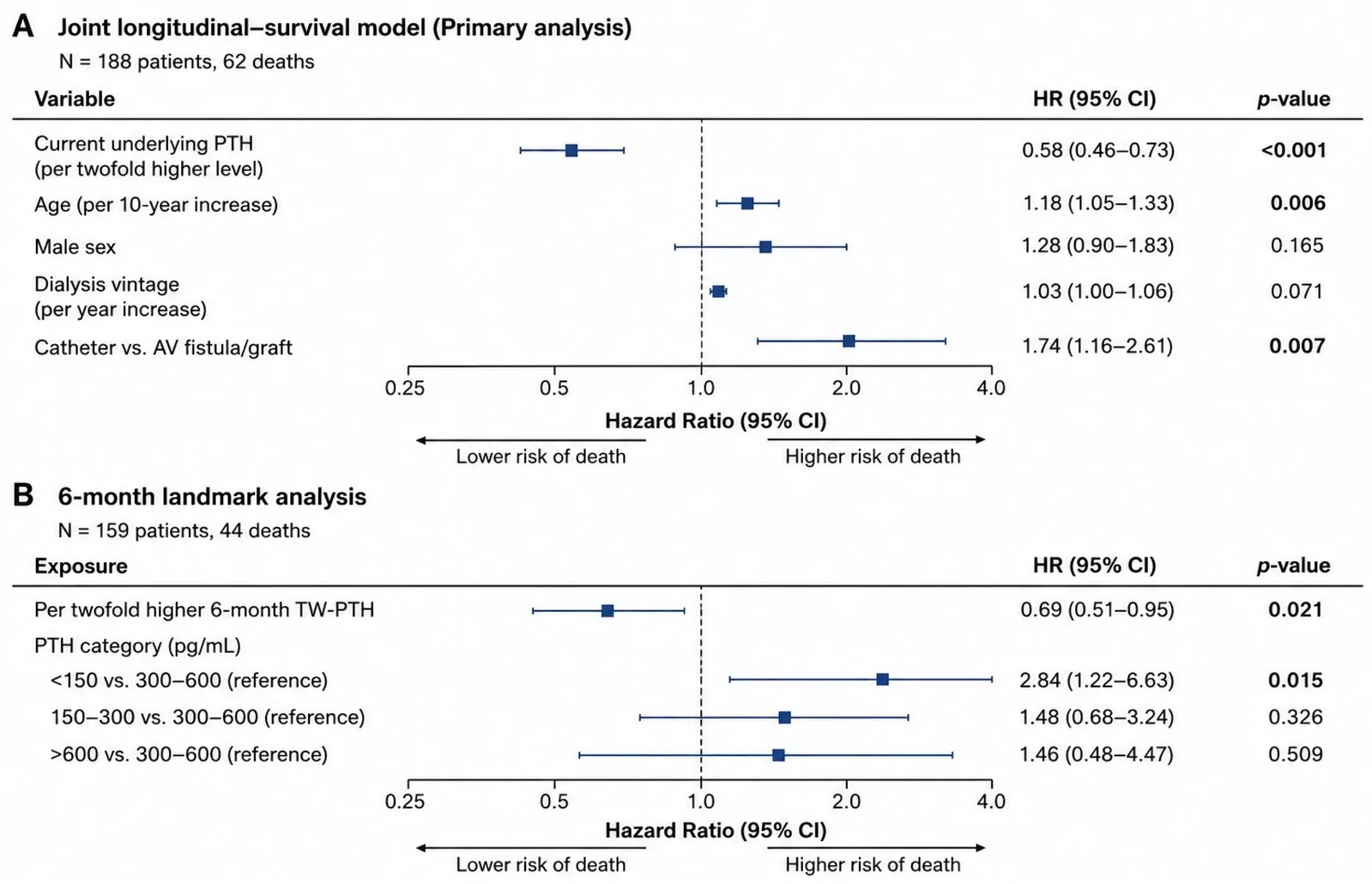
Primary and sensitivity analyses of the association between PTH and all-cause mortality. (**A**) Results of the primary joint longitudinal-survival model linking repeated log2-transformed parathyroid hormone (PTH) measurements with all-cause mortality. Hazard ratios (HRs) are adjusted for age, sex, dialysis vintage, and dialysis access. PTH is expressed per twofold higher estimated current underlying PTH level. (**B**) Results of the 6-month landmark analysis. Time-weighted mean PTH (TW-PTH) was calculated using measurements obtained during the first 6 months after the initial PTH measurement, and mortality follow-up began at the 6-month landmark among patients who remained alive and under follow-up at that time. Hazard ratios are presented per twofold higher TW-PTH and according to clinically relevant PTH categories, using 300–600 pg/mL as the reference group. Blue squares represent adjusted hazard ratios, and horizontal lines indicate 95% confidence intervals. Abbreviations: AV, arteriovenous; CI, confidence interval; HR, hazard ratio; PTH, parathyroid hormone; TW, time-weighted.

In the adjusted joint model, higher current underlying PTH levels were associated with lower observed mortality hazard. Each twofold higher estimated current PTH level was associated with a 42% lower observed mortality hazard (adjusted HR 0.58, 95% CI 0.46–0.73; *p* < 0.001) (Table 5 and Figure 3A). These findings indicate that lower estimated current underlying PTH levels were associated with higher observed mortality hazard, while accounting for the repeated longitudinal nature of PTH measurements.

#### 3.5.2. Sensitivity Analysis (Six-Month Landmark Analysis)

To establish temporal separation between PTH ascertainment and mortality follow-up, a post hoc 6-month landmark sensitivity analysis was performed. A total of 159 patients remained alive and under follow-up at the landmark, among whom 44 subsequent deaths occurred. Higher 6-month time-weighted mean PTH remained associated with lower subsequent mortality hazard, with each twofold higher PTH level associated with a 31% lower adjusted mortality hazard (adjusted HR 0.69, 95% CI 0.51–0.95; *p* = 0.021).

When analyzed according to clinically relevant PTH categories, patients with time-weighted mean PTH < 150 pg/mL had significantly higher mortality than those with PTH 300–600 pg/mL (adjusted HR 2.84, 95% CI 1.22–6.63; *p* = 0.015), whereas the estimates for the 150–300 pg/mL and >600 pg/mL categories were not statistically significant (Table 5 and Figure 3B). Similar findings were observed in sensitivity analyses restricted to patients with at least two PTH measurements during the ascertainment window and in analyses performed without carrying the final PTH value forward to the landmark, supporting the robustness of the observed association between low longitudinal PTH burden and mortality (Table 5).

#### 3.5.3. Secondary Descriptive Survival Analyses

During follow-up, 62 patients died, while 126 remained alive or were censored. Patients who died contributed fewer PTH measurements and had shorter PTH ascertainment windows than patients who remained alive or were censored. The median number of PTH measurements was 5 [IQR 2–10] among patients who died and 9 [IQR 4–15] among those who remained alive or were censored (*p* < 0.001). The corresponding median PTH observation windows were 281 [77–519] and 540 [189–1008] days, respectively (*p* < 0.001).

**Figure 4:**
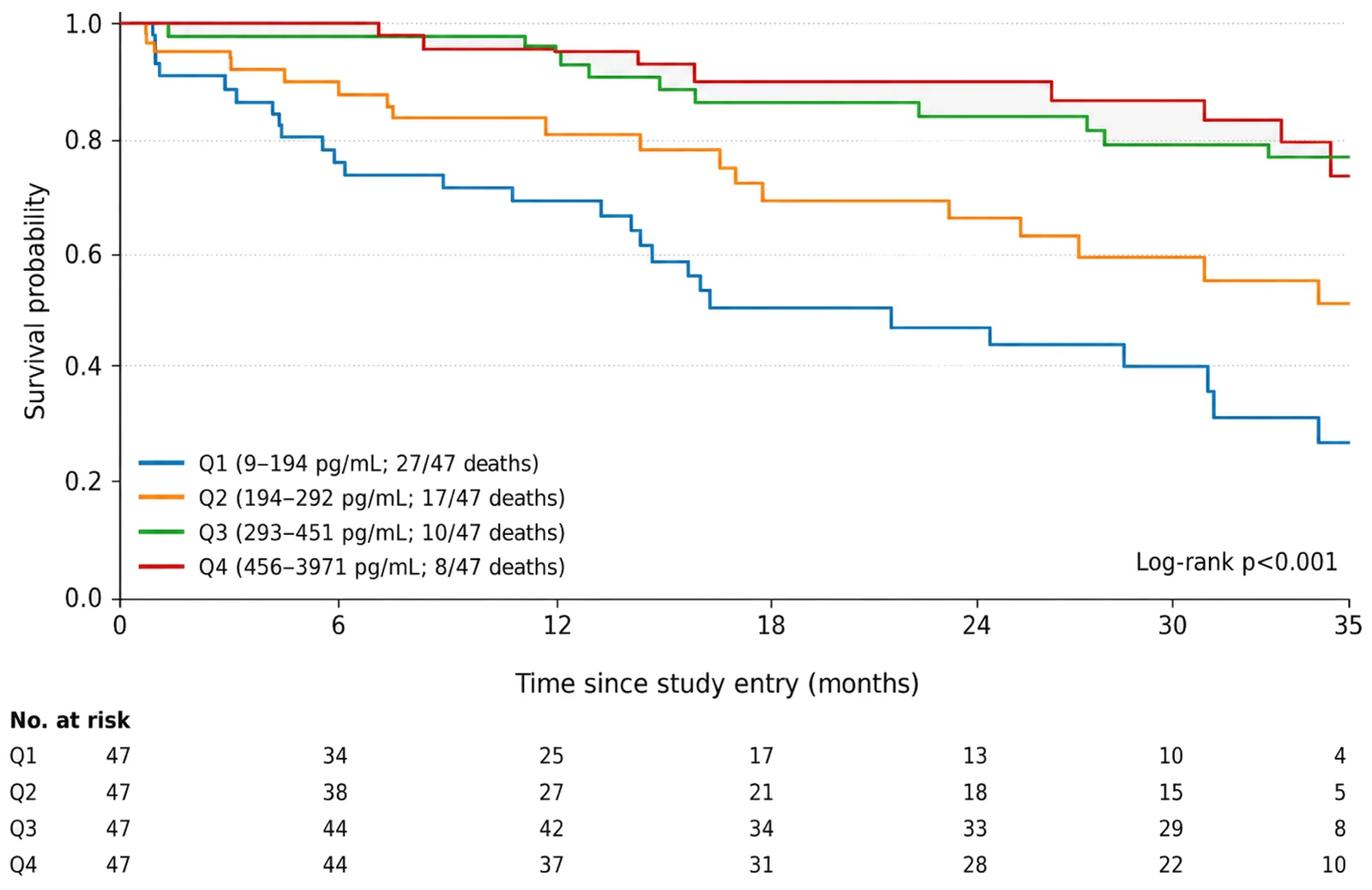
Kaplan–Meier estimates of overall survival according to quartiles of time-weighted mean PTH. Unadjusted Kaplan–Meier curves show observed survival during follow-up according to quartiles of time-weighted mean parathyroid hormone (PTH). Patients in the lowest PTH quartile (Q1) had the highest observed all-cause mortality and the lowest overall survival, whereas mortality was lower and survival was higher in the remaining quartiles. The global log-rank test indicated a significant difference between groups (*p* < 0.001). Numbers below the plot indicate the number of patients at risk at each time point. Time-weighted mean PTH was calculated from serial PTH measurements accumulated during follow-up. Because the PTH ascertainment window overlapped with mortality follow-up, these curves should be interpreted descriptively and not as estimates of baseline prognostic effects.

Conventional survival analyses based on time-weighted mean PTH accumulated throughout follow-up were performed as secondary descriptive analyses to facilitate comparison with previous studies and to characterize the observed longitudinal relationship between PTH burden and mortality. Kaplan–Meier curves demonstrated progressively lower survival among patients in the lowest quartile of time-weighted mean PTH (log-rank *p* < 0.001) (Figure 4).

Adjusted Cox proportional hazards models showed a consistent pattern across continuous, quartile-based, and clinically defined PTH categories (Table 6). Compared with patients with a time-weighted mean PTH of 300–600 pg/mL, those with PTH < 150 pg/mL had the highest observed mortality hazard (adjusted HR 5.88, 95% CI 2.72–12.71), whereas patients with PTH 150–300 pg/mL also had higher mortality (adjusted HR 3.05, 95% CI 1.45–6.38). The estimate for PTH > 600 pg/mL was imprecise (adjusted HR 1.49, 95% CI 0.52–4.28).

**Figure 5:**
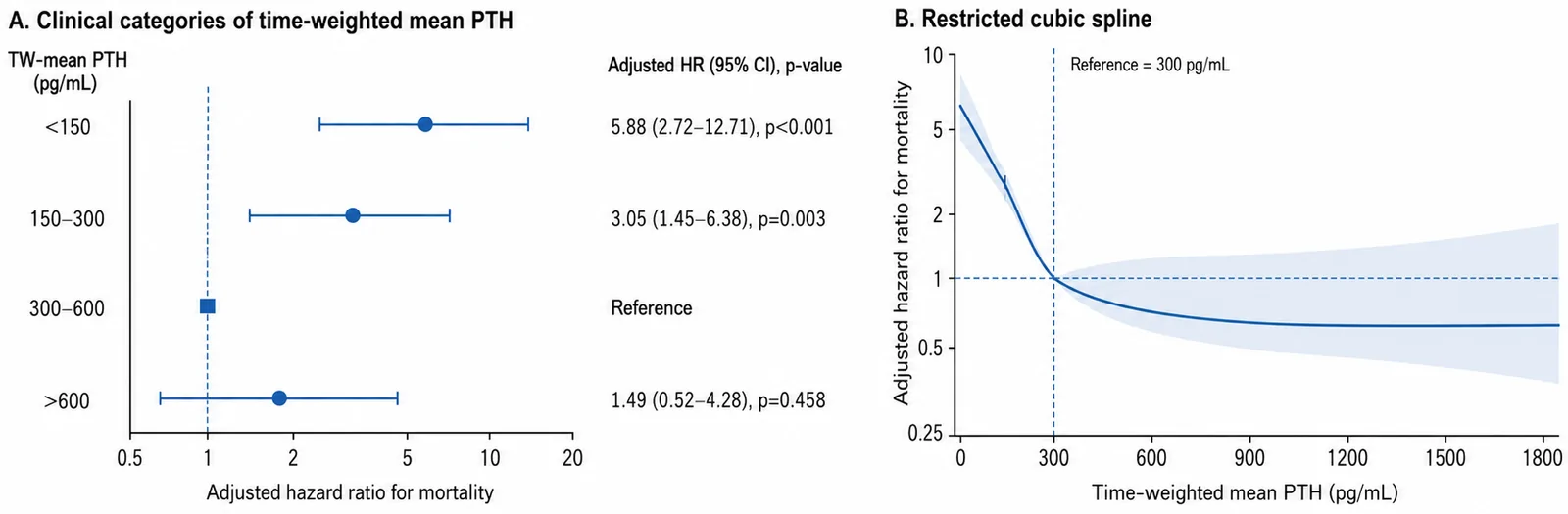
Adjusted association of time-weighted mean PTH with mortality. (**A**) Adjusted hazard ratios for all-cause mortality according to clinical categories of time-weighted mean parathyroid hormone (PTH), using 300–600 pg/mL as the reference category. Time-weighted mean PTH < 150 pg/mL and 150–300 pg/mL were associated with higher observed mortality hazards compared with the reference group. The estimate for PTH > 600 pg/mL was imprecise and did not permit a reliable determination of whether mortality hazard differed from that of the reference group. (**B**) Restricted cubic spline analysis of the adjusted association between time-weighted mean PTH and mortality hazard, with 300 pg/mL used as the reference value. The highest estimated mortality hazards were observed at low PTH levels, with a steep decline toward the reference value and progressively wider confidence intervals at higher PTH levels. The shaded area represents the 95% confidence interval. All models were adjusted for age, sex, dialysis vintage, and dialysis access. Because PTH measurements were accumulated during the mortality follow-up period, the estimates represent longitudinal associations and should not be interpreted as baseline prognostic or causal effects.

Restricted cubic spline analysis demonstrated a non-linear inverse association between longitudinal PTH burden and observed mortality hazard, with the highest estimated hazard at lower PTH levels and progressively lower hazard as PTH increased toward the recommended range (Figure 5). However, because time-weighted mean PTH was calculated from measurements accumulated during follow-up, these Cox and spline analyses should be interpreted as descriptive longitudinal association analyses rather than baseline prognostic models.

After excluding the 23 patients with only one available PTH measurement, 165 patients with 51 deaths remained. The continuous association remained statistically significant after adjustment for age, sex, dialysis vintage, and dialysis access (HR 0.86 per 100 pg/mL higher time-weighted mean PTH, 95% CI 0.75–0.99; *p* = 0.036). Compared with the 300–600 pg/mL reference group, the adjusted hazard ratios were 5.51 (95% CI 2.38–12.79; *p* < 0.001) for PTH < 150 pg/mL, 3.41 (95% CI 1.55–7.50; *p* = 0.002) for PTH 150–300 pg/mL, and 1.77 (95% CI 0.59–5.31; *p* = 0.309) for PTH >600 pg/mL (Table 6). These findings indicate that the category-based associations were not driven exclusively by patients with a single PTH measurement, although the analysis does not eliminate reverse causation or survivorship bias.

### 3.6. Sensitivity Analyses Using Arithmetic Mean PTH

To evaluate whether the method used to summarize longitudinal PTH influenced the findings, all hospitalization and mortality analyses were repeated using the arithmetic mean of serial PTH measurements. Overall, the results were highly consistent with those obtained using time-weighted mean PTH (Supplementary Table S1). Arithmetic mean PTH showed near-perfect agreement with time-weighted mean PTH (Pearson r = 0.999; Spearman ρ = 0.995), and 181 of 188 patients (96.3%) were classified into the same clinical PTH category using both approaches. Bland–Altman analysis demonstrated minimal average bias between the two summary measures, with a mean difference of −0.11 pg/mL and 95% limits of agreement from −51.15 to 50.93 pg/mL (Supplementary Figure S1).

Mortality and hospitalization analyses based on arithmetic mean PTH yielded effect estimates that were directionally consistent with those obtained using time-weighted mean PTH. These findings indicate that the observed associations were robust to the method used to summarize repeated PTH measurements.

## 4. Discussion

In this single-center retrospective cohort of patients receiving maintenance hemodialysis, low time-weighted PTH was associated with an adverse nutritional-inflammatory profile and greater functional dependence. In the primary mortality analysis, lower estimated current underlying PTH levels were associated with higher observed mortality hazard. The primary mortality analysis, based on a joint longitudinal-survival model linking repeated PTH measurements with all-cause mortality, demonstrated that lower current underlying PTH levels were associated with higher observed mortality hazard after adjustment for age, sex, dialysis vintage, and dialysis access.

The 6-month landmark analysis yielded directionally consistent findings, supporting temporal separation between PTH ascertainment and subsequent mortality follow-up. Conventional Cox analyses based on time-weighted mean PTH showed similar overall patterns and are presented as complementary descriptive analyses. Together, these findings support interpretation of persistently low PTH as part of a broader nutritional-inflammatory and functional phenotype rather than as an isolated marker of CKD-MBD control. Despite the temporal separation introduced by the landmark analysis, the observational design precludes causal inference. Low longitudinal PTH may therefore reflect underlying illness severity, inflammation, nutritional deterioration, treatment-related suppression, including prior suppression followed by discontinuation of calcimimetic or vitamin D therapy, or other processes evolving concurrently with adverse outcomes rather than acting as a direct determinant of mortality.

Interpretation of PTH in patients receiving maintenance hemodialysis has long been challenging. Contemporary CKD-MBD guidelines emphasize the importance of serial PTH trends and integrated assessment of calcium, phosphate, bone disease, and cardiovascular risk rather than reliance on a single biochemical threshold [1,4]. Accordingly, PTH should not be viewed solely as a surrogate marker of bone turnover but interpreted within the broader clinical context, where nutritional status, inflammation, comorbidity, CKD-MBD treatment, and functional reserve frequently coexist and influence both PTH levels and clinical outcomes.

In the present study, low longitudinal PTH burden was consistently associated with lower albumin, transferrin, and cholesterol concentrations together with higher inflammatory markers, supporting the concept that persistently low PTH identifies a vulnerable nutritional-inflammatory profile rather than simply reflecting apparently favorable CKD-MBD control.

The relationship between PTH and clinical outcomes is increasingly recognized to be complex and non-linear. Although elevated PTH has traditionally been regarded as a marker of high-turnover bone disease and has been associated with adverse outcomes in observational studies [5,7], randomized evidence has not clearly demonstrated that pharmacological PTH lowering improves hard clinical outcomes.

In the EVOLVE trial, cinacalcet did not significantly reduce death or major cardiovascular events in the primary intention-to-treat analysis [8]. Conversely, several observational studies have suggested that relatively low PTH levels may reflect malnutrition-inflammation rather than optimal mineral metabolism [6,9,10,11]. Our findings are consistent with this concept and extend previous observations by demonstrating that low longitudinal PTH burden clustered with adverse nutritional-inflammatory biomarkers in a real-world maintenance hemodialysis cohort.

These observations support interpretation of persistently low PTH as a contextual biomarker that reflects the combined effects of CKD-MBD, nutritional reserve, inflammation, and overall health status rather than as an isolated therapeutic target. This broader interpretation may be particularly relevant in patients receiving maintenance hemodialysis, in whom multiple interrelated biological processes evolve simultaneously. Whether low PTH itself contributes to these abnormalities or primarily reflects them cannot be determined from the present observational study.

An important finding of the present study is that the association between low longitudinal PTH burden and adverse nutritional-inflammatory biomarkers extended beyond laboratory abnormalities to routinely documented measures of physical function and independence. Higher time-weighted mean PTH remained independently associated with lower odds of impaired mobility and dependence in dressing, bathing, toileting, and continence after adjustment for age, sex, dialysis vintage, and dialysis access. These associations suggest that the biochemical profile accompanying low PTH was paralleled by clinically meaningful functional impairment.

Several mechanisms may explain these observations. Protein-energy wasting and chronic inflammation are major determinants of sarcopenia, frailty, and functional decline in patients receiving maintenance hemodialysis [12]. Although direct assessments of body composition, muscle mass, muscle strength, dietary intake, and validated frailty measures were unavailable, the combination of lower albumin, lower cholesterol, higher inflammatory markers, and greater dependence in activities of daily living is consistent with a vulnerable clinical phenotype characterized by reduced physiological reserve rather than isolated disturbances of mineral metabolism.

Our findings also extend previous studies linking low PTH with malnutrition-inflammation. Dukkipati et al. demonstrated that relatively low PTH levels were associated with malnutrition-inflammation complex syndrome and survival [6], while Feroze et al. similarly suggested that low PTH may reflect nutritional and inflammatory derangements rather than optimal CKD-MBD control [10].

More recently, Zilberman-Itskovich et al. reported longitudinal associations between low PTH and adverse nutritional-inflammatory characteristics in maintenance hemodialysis patients [13]. The present study extends these observations by demonstrating that this unfavorable biochemical profile is accompanied by routinely documented functional dependence, thereby linking longitudinal PTH burden with clinically relevant measures of patient independence in routine dialysis care.

Functional decline is highly relevant in maintenance hemodialysis but is infrequently incorporated into studies of CKD-MBD. In a large DOPPS analysis, Kanda et al. showed that the combination of malnutrition-inflammation and functional limitations was associated with substantially higher mortality [14]. Other studies have likewise linked CKD-MBD and malnutrition-inflammation with impaired health-related quality of life in patients receiving hemodialysis [15,16,17,18].

Our findings complement this literature by suggesting that persistently low longitudinal PTH may identify patients in whom nutritional vulnerability, inflammation, and functional dependence coexist. These observations support a broader clinical assessment of patients with persistently low PTH rather than interpretation of low PTH as an isolated indicator of successful CKD-MBD control.

The mortality analyses further support an association between lower PTH levels and a clinically vulnerable phenotype of patients receiving maintenance hemodialysis. Unlike conventional Cox models based on summary PTH measures, the primary analysis used a joint longitudinal–survival model that simultaneously incorporated repeated PTH measurements and survival, thereby accounting for the longitudinal evolution of PTH and reducing bias arising from informative termination of longitudinal biomarker measurements by death. This analysis demonstrated that lower current underlying PTH levels were associated with higher observed mortality hazard after adjustment for age, sex, dialysis vintage, and dialysis access. Although the observational design precludes causal inference, these findings strengthen the evidence that the association between persistently low PTH and mortality is not simply attributable to differences in the number or timing of available PTH measurements.

The 6-month landmark analysis provided additional support for the robustness of this association by separating the PTH ascertainment period from subsequent mortality follow-up. Similar results were observed after restricting the cohort to patients with at least two PTH measurements during the ascertainment period and in analyses performed without carrying the final PTH value forward to the landmark. Together, these complementary analyses reduce concerns regarding reverse causation and survivorship bias, although they cannot eliminate residual confounding or establish causality.

Our findings are also consistent with previous observational studies reporting a non-linear relationship between PTH and adverse outcomes. Large cohort studies and meta-analyses have associated disturbances in PTH, calcium, and phosphate with mortality among patients receiving dialysis [5,7], whereas the EVOLVE trial did not demonstrate a significant reduction in death or major cardiovascular events with cinacalcet in the primary intention-to-treat analysis [8]. Rather than supporting a simple “lower is better” interpretation of PTH, these data suggest that both markedly elevated and persistently low PTH may reflect distinct high-risk clinical states.

In the present study, the highest observed mortality hazard was concentrated among patients with persistently low longitudinal PTH burden, whereas the estimate for PTH >600 pg/mL remained imprecise because of the limited number of patients and events in this category. Accordingly, our findings should not be interpreted as evidence that high PTH is benign, but rather that persistently low PTH may identify a subgroup of patients with particularly high clinical vulnerability.

Several mechanisms may contribute to these observations. Low PTH may accompany protein-energy wasting, chronic inflammation, reduced physiological reserve, treatment-related suppression of the parathyroid axis, or low bone turnover and adynamic bone disease, particularly in older or medically complex patients [19]. These mechanisms are not mutually exclusive and could coexist within the same patient. Because detailed longitudinal treatment data, bone turnover markers, and bone histology were unavailable, the present study cannot distinguish among these possibilities. The observed associations should therefore be interpreted as reflecting a broader clinical phenotype rather than a specific biological pathway.

The present findings also have important clinical implications for the interpretation of PTH in maintenance hemodialysis. Our results should not be interpreted as suggesting that suppression of secondary hyperparathyroidism is undesirable or that high PTH is clinically benign. Previous studies have shown that severe and sustained secondary hyperparathyroidism may contribute to weight loss, nutritional deterioration, and adverse outcomes. In a large DOPPS analysis, Komaba et al. reported that secondary hyperparathyroidism was associated with subsequent weight loss and higher long-term mortality [20]. Similarly, Disthabanchong et al. demonstrated associations between severe secondary hyperparathyroidism and impaired nutritional status [21], while nutritional parameters improved following parathyroidectomy in patients with severe disease [22]. Rather than being contradictory, these findings suggest that the clinical significance of PTH depends on the broader biological and clinical context.

Taken together, the available evidence supports a U-shaped conceptual framework in which both persistently elevated and persistently low PTH may identify vulnerable but biologically distinct patient populations. Severe hyperparathyroidism may reflect uncontrolled CKD-MBD with high bone turnover and catabolic consequences, whereas persistently low PTH may accompany protein-energy wasting, chronic inflammation, reduced physiological reserve, low bone turnover, or treatment-related suppression of the parathyroid axis. Accordingly, interpretation of PTH should extend beyond numerical treatment targets and incorporate nutritional status, inflammatory burden, functional dependence, CKD-MBD therapy, and the overall clinical condition of the patient.

From a practical perspective, persistently low PTH should not automatically be interpreted as evidence of successful CKD-MBD management. Instead, clinicians should consider whether low PTH coexists with biochemical evidence of nutritional deterioration, chronic inflammation, or functional decline. Such patients may benefit from a broader multidisciplinary assessment of nutritional intake, weight trajectory, muscle function, frailty, inflammatory status, and CKD-MBD treatment intensity rather than isolated adjustment of PTH-directed therapy. Although our findings do not establish that modifying PTH itself improves outcomes, they suggest that persistently low longitudinal PTH may serve as a useful clinical signal prompting more comprehensive patient evaluation.

This study has several limitations. First, it was a retrospective single-center study with a relatively modest sample size, particularly in the extreme PTH categories, resulting in limited precision of some category-specific estimates. Accordingly, the wide confidence intervals, especially for patients with PTH > 600 pg/mL, preclude firm conclusions regarding the magnitude of risk in this subgroup.

Second, although the primary mortality analysis used a joint longitudinal-survival model and the 6-month landmark analysis established temporal separation between PTH ascertainment and subsequent mortality follow-up, the observational design cannot establish causality. Residual confounding remains possible despite adjustment for age, sex, dialysis vintage, and dialysis access. Important variables such as overall comorbidity burden, cardiovascular disease severity, residual kidney function, detailed nutritional status, body composition, dietary intake, chronic inflammatory disorders, and validated frailty measures were unavailable or incompletely recorded. Consequently, low longitudinal PTH should be interpreted as a marker associated with an adverse clinical phenotype rather than an independent causal determinant of mortality.

Third, CKD-MBD therapies were not included in the adjusted models because treatment decisions are largely driven by PTH, calcium, and phosphate levels and therefore may represent downstream variables. In addition, only current CKD-MBD medication use was available. Longitudinal treatment histories, including treatment initiation, discontinuation, and dose adjustments, were unavailable. Consequently, we could not determine whether low PTH reflected spontaneous biological variation or previous treatment-induced suppression followed by dose reduction or treatment discontinuation in accordance with contemporary clinical practice guidelines. This limitation precluded assessment of cumulative treatment exposure or treatment sequencing and may have contributed to the observed associations between low PTH and clinical outcomes.

Fourth, direct assessments of bone turnover, including bone-specific alkaline phosphatase, other bone turnover markers, fracture outcomes, vascular calcification, and bone biopsy, were unavailable. Similarly, functional outcomes were derived from routinely documented mobility and activities-of-daily-living assessments rather than validated frailty, sarcopenia, physical-performance, or patient-reported quality-of-life instruments. These measures provide clinically relevant information regarding day-to-day functional dependence but may be less precise and less comparable with standardized research instruments.

Functional assessments were not available for all participants because these variables were extracted from routine clinical documentation, and functional analyses were therefore performed using available-case data without imputation. Importantly, missingness was not uniform across PTH categories and was more frequent among patients with lower time-weighted mean PTH. Because differential documentation may have introduced selection or information bias, the functional findings should therefore be interpreted cautiously and require confirmation in prospective studies using standardized functional assessments. In addition, the whole-follow-up time-weighted PTH summary was not temporally aligned with the biochemical and functional assessments and could include PTH measurements obtained after these assessments. Accordingly, these analyses should be interpreted as descriptive associations between longitudinal PTH burden and the corresponding clinical measures, without inference regarding temporal directionality.

Finally, although false discovery rate correction was applied to the prespecified biomarker and functional-outcome analyses, these analyses remain exploratory and hypothesis-generating. The consistency of the findings across multiple complementary analytical approaches—including the joint longitudinal-survival model, landmark analysis, conventional Cox analyses, and sensitivity analyses using arithmetic mean PTH—supports the robustness of the observed associations but does not eliminate the possibility of residual confounding or chance findings.

Future prospective multicenter studies incorporating predefined PTH ascertainment periods, time-varying treatment data, validated frailty and sarcopenia assessments, objective body composition measurements, and bone turnover markers are needed to determine the prognostic significance and biological mechanisms underlying persistently low PTH in maintenance hemodialysis.

Despite these limitations, the present study has several important strengths. First, repeated PTH measurements were available over an extended follow-up period, allowing evaluation of longitudinal PTH burden rather than reliance on a single baseline measurement. Second, the primary mortality analysis used a joint longitudinal-survival model that accounted for the longitudinal evolution of PTH and informative termination of repeated measurements by death, while a post hoc 6-month landmark sensitivity analysis provided complementary support for temporal separation between PTH ascertainment and subsequent mortality. Third, nutritional-inflammatory biomarkers, routinely documented functional measures, hospitalization burden, and mortality were evaluated within the same cohort, providing an integrated assessment of the clinical profile associated with longitudinal PTH burden. Finally, the consistency of the findings across the primary joint model, landmark analysis, conventional Cox analyses, and sensitivity analyses using arithmetic mean PTH supports the robustness of the overall observations, although it does not establish causality or eliminate residual confounding.

## 5. Conclusions

In conclusion, low time-weighted PTH in patients receiving maintenance hemodialysis was associated with an adverse nutritional-inflammatory profile and greater functional dependence. In the primary joint longitudinal-survival model, lower estimated current underlying PTH levels were associated with higher mortality hazard. The 6-month landmark analysis and secondary descriptive Cox analyses using time-weighted mean PTH yielded directionally consistent findings. These findings support interpretation of persistently low PTH as part of a broader nutritional-inflammatory and functional phenotype rather than as an isolated indicator of CKD-MBD control. Because of the observational design, these associations should not be interpreted as causal or as evidence that modifying PTH itself improves outcomes. Instead, persistently low PTH may serve as a clinically useful signal prompting a comprehensive assessment of nutritional status, inflammatory burden, functional decline, and CKD-MBD management. Prospective multicenter studies incorporating longitudinal treatment data, validated frailty assessments, body composition measurements, and direct markers of bone turnover are needed to clarify the biological mechanisms and prognostic significance of persistently low PTH in maintenance hemodialysis.

## Figures and Tables

**Table 1:** Patient characteristics according to time-weighted mean PTH categories.

Variable	Overall (N = 188)	<150 (n = 33)	150–300 (n = 64)	300–600 (n = 64)	>600 (n = 27)	*p*-Value
Demographic and dialysis characteristics						
Age, years	70 [59–77]	70 [62–79]	71 [65–80]	71 [62–76]	58 [46–69]	<0.001
Male sex, n (%)	128 (68.1)	26 (78.8)	41 (64.1)	45 (70.3)	16 (59.3)	0.343
Time from initiation of hemodialysis at study unit to first PTH, months	2.9 [0.5–17.9]	1.1 [0.0–11.2]	2.0 [0.5–11.2]	6.8 [1.1–21.9]	6.4 [1.4–39.4]	0.041
PTH observation period, days	424 [145–919]	187 [3–464]	248 [86–726]	815 [371–1008]	637 [190–1008]	<0.001
Number of PTH measurements	7 [4–13]	4 [2–8]	5 [2–12]	11 [7–16]	11 [4–16]	<0.001
Catheter vascular access, n (%)	130 (69.1)	25 (75.8)	51 (79.7)	33 (51.6)	21 (77.8)	0.001
Kt/V	1.30 [1.10–1.50]	1.27 [1.00–1.40]	1.40 [1.10–1.60]	1.30 [1.10–1.50]	1.38 [1.10–1.60]	0.413
Dry weight, kg	70.5 [59.0–81.0]	71.0 [58.0–79.0]	67.5 [58.8–78.5]	69.5 [57.5–83.0]	77.5 [68.9–84.9]	0.282
Dialysis sessions/week	3 [3–3]	3 [3–3]	3 [3–3]	3 [3–3]	3 [3–3]	0.690
Session duration, hours	3.5 [3.0–4.0]	3.5 [3.0–4.0]	3.5 [3.0–4.0]	4.0 [3.5–4.0]	4.0 [3.5–4.0]	0.024
Weekly dialysis time, hours	10.5 [9.0–12.0]	10.5 [9.0–12.0]	10.5 [9.0–12.0]	12.0 [10.2–12.0]	12.0 [10.5–12.0]	0.074
CKD-MBD treatment						
Phosphate binder use, n (%)	160 (85.1)	26 (78.8)	48 (75.0)	62 (96.9)	24 (88.9)	0.004
Vitamin D derivative use, n (%)	152 (80.9)	24 (72.7)	45 (70.3)	59 (92.2)	24 (88.9)	0.006
Cinacalcet use, n (%)	50 (26.6)	1 (3.0)	10 (15.6)	29 (45.3)	10 (37.0)	<0.001
Etelcalcetide use, n (%)	12 (6.4)	0 (0.0)	0 (0.0)	0 (0.0)	12 (44.4)	<0.001
Calcium therapy, n (%)	103 (54.8)	18 (54.5)	31 (48.4)	40 (62.5)	14 (51.9)	0.445
PTH exposure and variability						
Arithmetic mean PTH, pg/mL	292 [202–459]	109 [91–133]	233 [204–264]	407 [339–473]	884 [740–1346]	<0.001
Time-weighted mean PTH, pg/mL	292 [194–453]	109 [84–133]	232 [200–261]	404 [338–475]	905 [727–1340]	<0.001
PTH SD, pg/mL	83 [38–156]	32 [4–50]	61 [22–97]	119 [81–187]	331 [212–430]	<0.001
PTH coefficient of variation, %	29.0 [17.3–43.3]	33.7 [10.3–46.5]	28.6 [8.7–39.7]	28.7 [20.0–45.7]	27.9 [19.8–39.0]	0.494
Time in PTH target range 150–600, %	80.9 [23.9–100.0]	0.0 [0.0–20.9]	100.0 [83.8–100.0]	96.0 [78.3–100.0]	5.8 [0.0–25.5]	<0.001
Time above PTH 600, %	0.0 [0.0–10.3]	0.0 [0.0–0.0]	0.0 [0.0–0.0]	3.2 [0.0–16.8]	94.2 [74.5–100.0]	<0.001
CKD-MBD biochemical parameters						
Serum calcium, mg/dL	8.6 [8.0–9.0]	8.3 [7.9–9.1]	8.5 [8.0–9.0]	8.6 [8.3–8.9]	8.7 [7.8–9.0]	0.869
Serum phosphate, mg/dL	5.4 [4.7–6.6]	5.1 [4.1–5.9]	5.2 [4.3–6.2]	5.5 [4.8–6.5]	6.8 [5.7–8.2]	<0.001

Abbreviations: AVF, arteriovenous fistula; AVG, arteriovenous graft; CKD-MBD, chronic kidney disease-mineral and bone disorder; IQR, interquartile range; PTH, parathyroid hormone; SD, standard deviation. Continuous variables are presented as median [IQR]; categorical variables as n (%). *p*-values were calculated using Kruskal–Wallis tests for continuous variables and chi-square or Fisher exact tests for categorical variables, as appropriate. Time-weighted mean PTH was calculated using linear interpolation between consecutive PTH measurements. Patients with one PTH measurement were assigned that single value as their time-weighted mean PTH.

**Table 2:** Nutritional-inflammatory biomarker distributions according to clinical categories of time-weighted mean PTH.

Biomarker	<150	150–300	300–600	>600	*p*-Value	FDR q-Value
**Albumin**	3.40 [2.70–3.70]	3.54 [3.21–4.00]	3.80 [3.50–4.14]	4.00 [3.65–4.16]	<0.001	0.002
**Phosphorus**	5.10 [4.10–5.90]	5.20 [4.33–6.22]	5.55 [4.80–6.50]	6.80 [5.70–8.25]	<0.001	0.002
**Cholesterol**	114 [91–139]	134 [106–157]	132 [106–162]	150 [133–168]	0.019	0.019
**Transferrin**	146 [107–170]	148 [125–178]	158 [145–182]	180 [158–206]	<0.001	0.002
**Ferritin**	507 [314–1182]	611 [338–1178]	504 [275–756]	351 [185–510]	0.009	0.014
**CRP**	25.0 [11.5–63.0]	11.1 [4.8–47.7]	10.0 [2.7–34.0]	9.5 [4.1–16.9]	0.018	0.019

Abbreviations: PTH, parathyroid hormone; IQR, interquartile range; FDR, false discovery rate; CRP, *C*-reactive protein. Values are presented as median [IQR]. PTH categories are based on time-weighted mean PTH (<150, 150–300, 300–600, and >600 pg/mL). *p*-values were obtained using the Kruskal–Wallis test. False discovery rate-adjusted q-values were calculated using the Benjamini–Hochberg procedure within the corresponding family of analyses.

**Table 3:** Continuous associations between time-weighted mean PTH and nutritional-inflammatory biomarkers.

Biomarker	N	β per 100 pg/mL Higher TW-Mean PTH	95% CI	*p*-Value	FDR q-Value
**Nutritional and inflammatory biomarkers**					
Hemoglobin	187	−0.018	−0.071 to 0.035	0.516	0.631
Calcium	186	−0.006	−0.032 to 0.020	0.725	0.798
Phosphorus	186	0.116	0.063 to 0.170	<0.001	<0.001
Albumin	186	0.040	0.022 to 0.058	<0.001	<0.001
Cholesterol	176	1.667	0.239 to 3.095	0.023	0.064
Triglycerides	176	1.870	−0.964 to 4.703	0.183	0.252
Iron	176	0.678	−0.107 to 1.463	0.085	0.156
Transferrin	184	2.320	1.081 to 3.560	<0.001	0.001
Ferritin	179	−0.033	−0.0004 to −0.066	0.048	0.102
CRP	184	−0.030	−0.074 to 0.013	0.172	0.252

Abbreviations: CI, confidence interval; CRP, *C*-reactive protein; FDR, false discovery rate; PTH, parathyroid hormone; TW, time-weighted. β values represent unadjusted linear regression coefficients per 100 pg/mL higher time-weighted mean PTH. Positive β values indicate higher biomarker values with increasing PTH, whereas negative β values indicate lower biomarker values. For CRP and ferritin, regression analyses were performed using log-transformed biomarker values because of right-skewed distributions; therefore, β coefficients for these biomarkers are expressed on the transformed scale (log1p for CRP and natural logarithm for ferritin).

**Table 4:** Associations between time-weighted mean PTH and mobility and functional-dependence outcomes.

Outcome	N	Impaired/Worse Status, n (%)	Unadjusted OR per 100 pg/mL	*p*-Value	Adjusted OR per 100 pg/mL	*p*-Value	FDR q-Value
Mobility-related outcomes							
Gait instability	160	95 (59.4%)	0.87 (0.78–0.96)	0.008	0.91 (0.82–1.01)	0.079	0.105
Mobility aids use	160	91 (56.9%)	0.88 (0.79–0.97)	0.012	0.92 (0.82–1.02)	0.105	0.120
Worse mobility category	161	86 (53.4%)	0.78 (0.68–0.89)	<0.001	0.81 (0.70–0.93)	0.004	0.016
Activities of daily living							
Assistance with dressing	158	75 (47.5%)	0.76 (0.65–0.89)	0.001	0.81 (0.69–0.95)	0.008	0.018
Assistance with bathing	158	82 (51.9%)	0.77 (0.66–0.89)	0.001	0.82 (0.71–0.95)	0.009	0.018
Worse toileting function	159	65 (40.9%)	0.70 (0.58–0.84)	<0.001	0.73 (0.60–0.88)	0.001	0.008
Worse continence status	163	40 (24.5%)	0.78 (0.64–0.94)	0.010	0.80 (0.66–0.98)	0.029	0.046
Falls							
Falls history	149	25 (16.8%)	0.79 (0.62–1.00)	0.053	0.84 (0.66–1.05)	0.131	0.131

Abbreviations: OR, odds ratio; FDR, false discovery rate; PTH, parathyroid hormone. Odds ratios (ORs) represent the association per 100 pg/mL higher time-weighted mean PTH. Adjusted models included age, sex, dialysis vintage, and dialysis access. For ordinal outcomes, ORs represent the odds of belonging to a worse functional category. False discovery rate-adjusted q-values were calculated using the Benjamini–Hochberg procedure.

**Table 5:** Mortality analyses using repeated PTH measurements and time-weighted mean PTH.

Analysis	Exposure	N (Deaths)	Adjusted HR (95% CI)	*p*-Value
Primary analysis				
Joint longitudinal-survival model	Current underlying PTH (per twofold higher level)	188 (62)	0.58 (0.46–0.73)	<0.001
Sensitivity analysis				
6-month landmark	Per twofold higher 6-month TW-PTH	159 (44)	0.69 (0.51–0.95)	0.021
	PTH < 150 vs. 300–600 pg/mL	159 (44)	2.84 (1.22–6.63)	0.015
	PTH 150–300 vs. 300–600 pg/mL	159 (44)	1.48 (0.68–3.24)	0.326
	PTH > 600 vs. 300–600 pg/mL	159 (44)	1.46 (0.48–4.47)	0.509
Additional sensitivity analyses				
Landmark (≥2 PTH measurements)	Per twofold higher 6-month TW-PTH	149 (40)	0.72 (0.52–1.00)	0.048
	PTH < 150 vs. 300–600 pg/mL	149 (40)	2.65 (1.10–6.42)	0.031
Landmark (no carry-forward)	Per twofold higher 6-month TW-PTH	159 (44)	0.71 (0.52–0.97)	0.031
	PTH < 150 vs. 300–600 pg/mL	159 (44)	2.90 (1.22–6.91)	0.016
Secondary descriptive analyses				
Conventional Cox model	Per 100 pg/mL higher TW-PTH	188 (62)	0.83 (0.72–0.95)	0.007
	PTH < 150 vs. 300–600 pg/mL	188 (62)	5.88 (2.72–12.71)	<0.001
	PTH 150–300 vs. 300–600 pg/mL	188 (62)	3.05 (1.45–6.38)	0.003
	PTH > 600 vs. 300–600 pg/mL	188 (62)	1.49 (0.52–4.28)	0.463

Abbreviations: CI, confidence interval; HR, hazard ratio; PTH, parathyroid hormone; TW, time-weighted. The primary mortality analysis was performed using a joint longitudinal-survival model linking repeated log2-transformed PTH measurements with all-cause mortality. Hazard ratios for PTH are expressed per twofold higher estimated current underlying PTH level. A 6-month landmark analysis used time-weighted mean PTH calculated during the first 6 months after the initial PTH measurement, with mortality follow-up beginning at the landmark. Additional sensitivity analyses included restriction to patients with at least two PTH measurements and analysis without carrying the final PTH value forward to the landmark. Conventional Cox proportional hazards models based on time-weighted mean PTH accumulated throughout follow-up are presented as secondary descriptive analyses. Adjusted models included age, sex, dialysis vintage, and dialysis access. Hazard ratios are presented per twofold higher PTH level for the joint longitudinal–survival and landmark analyses, and per 100 pg/mL higher PTH for the conventional Cox analyses, consistent with the respective model specifications.

**Table 6:** Secondary descriptive Cox proportional hazards analyses according to longitudinal time-weighted mean PTH.

Analysis	Model	N	Deaths	HR (95% CI)	*p*-Value
Continuous time-weighted mean PTH					
Per 100 pg/mL higher TW-mean PTH	Unadjusted	188	62	0.81 (0.70–0.93)	0.003
Per 100 pg/mL higher TW-mean PTH	Adjusted	188	62	0.83 (0.72–0.95)	0.007
Quartiles of time-weighted mean PTH					
Q1	Reference	47	27	Reference	
Q2 vs. Q1	Adjusted	47	17	0.48 (0.26–0.89)	0.019
Q3 vs. Q1	Adjusted	47	10	0.21 (0.10–0.46)	<0.001
Q4 vs. Q1	Adjusted	47	8	0.20 (0.09–0.45)	<0.001
Clinical categories of time-weighted mean PTH					
300–600 pg/mL	Reference	64	11	Reference	
<150 pg/mL vs. 300–600 pg/mL	Adjusted	33	20	5.88 (2.72–12.71)	<0.001
150–300 pg/mL vs. 300–600 pg/mL	Adjusted	64	25	3.05 (1.45–6.38)	0.003
>600 pg/mL vs. 300–600 pg/mL	Adjusted	27	6	1.49 (0.52–4.28)	0.458
Sensitivity analysis excluding patients with one PTH measurement					
Per 100 pg/mL higher TW-mean PTH	Adjusted	165	51	0.86 (0.75–0.99)	0.036
<150 vs. 300–600 pg/mL	Adjusted	165	51	5.51 (2.38–12.79)	<0.001
150–300 vs. 300–600 pg/mL	Adjusted	165	51	3.41 (1.55–7.50)	0.002
>600 vs. 300–600 pg/mL	Adjusted	165	51	1.77 (0.59–5.31)	0.309

Abbreviations: CI, confidence interval; HR, hazard ratio; PTH, parathyroid hormone; Q, quartile; TW, time-weighted. Hazard ratios were estimated using Cox proportional hazards regression. Time-weighted mean parathyroid hormone (PTH) was analyzed continuously per 100 pg/mL higher PTH, by quartiles, and according to the clinical categories <150, 150–300, 300–600, and >600 pg/mL. Adjusted models included age, sex, dialysis vintage, and dialysis access. The lowest quartile (Q1) was the reference group in quartile-based analyses, and PTH 300–600 pg/mL was the reference group in clinical-category analyses. Sensitivity analyses excluded patients with only one available PTH measurement. Because time-weighted mean PTH was calculated from measurements accumulated during the mortality follow-up period, the estimates represent associations with a longitudinal PTH summary and should not be interpreted as baseline prognostic or causal effects. For the sensitivity-analysis rows, N and deaths refer to the overall analysis sample.

## Data Availability

The data that support the findings of this study are not publicly available because they contain information that could compromise participant privacy but may be available from the corresponding author upon reasonable request and subject to institutional approval.

## Supplementary Materials

The following supporting information can be downloaded at: https://www.mdpi.com/article/10.3390/nu18162742/s1, Figure S1. Bland-Altman comparison of arithmetic and time-weighted mean parathyroid hormone (PTH). Each point represents one patient. The x-axis shows the mean of the arithmetic and time-weighted mean PTH values, and the y-axis shows the difference between the time-weighted and arithmetic mean PTH values. The solid horizontal line indicates the mean difference (−0.11 pg/mL), and the dashed horizontal lines indicate the 95% limits of agreement; Table S1. Sensitivity analyses using arithmetic mean PTH instead of time-weighted mean PTH; Table S2. Missingness of functional assessments according to time-weighted mean PTH categories.
