# Supplementary material for: Low Time-Weighted Parathyroid Hormone Is Associated with Malnutrition-Inflammation and Functional Dependence in Maintenance Hemodialysis
Source: Nutrients. 2026 Aug 21;18(16):2742. doi: 10.3390/nu18162742 (PMC13516352; doi:10.3390/nu18162742)
Supplement: Supplementary file 1 [file nutrients-18-02742-s001.zip › nutrients-4420892-supplementary.pdf]

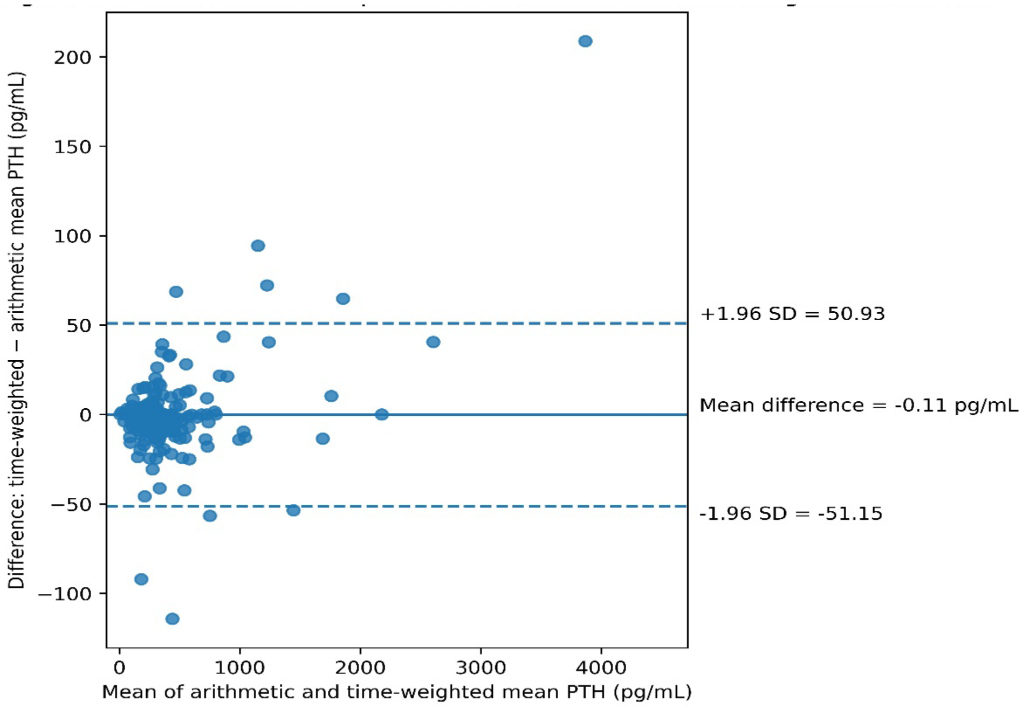

Mean difference = -0.11 pg/mL; 95% limits of agreement = -51.15 to 50.93 pg/mL; n = 188

Supplementary Figure S1. Bland-Altman comparison of arithmetic and time-weighted mean parathyroid hormone (PTH). Each point represents one patient. The x-axis shows the mean of the arithmetic and time-weighted mean PTH values, and the y-axis shows the difference between the time-weighted and arithmetic mean PTH values. The solid horizontal line indicates the mean difference (-0.11 pg/mL), and the dashed horizontal lines indicate the 95% limits of agreement.

Supplementary Table S1. Sensitivity analyses using arithmetic mean PTH instead of time-weighted mean PTH

Analysis	Exposure or comparison	N (deaths/events)	Effect estimate (95% CI)	p-value
**Continuous association with all-cause mortality**				
Cox proportional hazards model	Per 100 pg/mL higher arithmetic mean PTH; unadjusted	188 (62)	HR 0.80 (0.69-0.92)	0.003
Cox proportional hazards model	Per 100 pg/mL higher arithmetic mean PTH; adjusted	188 (62)	HR 0.82 (0.71-0.95)	0.006
**Clinical PTH categories and all-cause mortality**				
Cox proportional hazards model	<150 vs 300-600 pg/mL	30 (19) vs 64 (12)	HR 5.34 (2.51-11.35)	<0.001
Cox proportional hazards model	150-300 vs 300-600 pg/mL	67 (25) vs 64 (12)	HR 2.44 (1.20-4.94)	0.013
Cox proportional hazards model	>600 vs 300-600 pg/mL	27 (6) vs 64 (12)	HR 1.29 (0.46-3.63)	0.626
**Arithmetic mean PTH quartiles and all-cause mortality**				
Cox proportional hazards model	Q2 vs Q1	47 (16) vs 47 (28)	HR 0.40 (0.21-0.74)	0.004
Cox proportional hazards model	Q3 vs Q1	47 (9) vs 47 (28)	HR 0.18 (0.08-0.39)	<0.001
Cox proportional hazards model	Q4 vs Q1	47 (9) vs 47 (28)	HR 0.22 (0.10-0.48)	<0.001
**Hospitalization burden**				
Pearson correlation	Arithmetic mean PTH and hospitalization count	188	r = −0.051	0.491
Spearman correlation	Arithmetic mean PTH and hospitalization count	188	ρ = −0.056	0.447
Poisson hospitalization-rate model	Per 100 pg/mL higher arithmetic mean PTH; unadjusted	188	IRR 0.96 (0.92-1.00)	0.074
Poisson hospitalization-rate model	Per 100 pg/mL higher arithmetic mean PTH; adjusted	188	IRR 0.98 (0.94-1.00)	0.083

Abbreviations: CI, confidence interval; HR, hazard ratio; IRR, incidence rate ratio; PTH, parathyroid hormone; Q, quartile. Continuous estimates are expressed per 100 pg/mL higher arithmetic mean PTH. The 300-600 pg/mL category and Q1 were the reference groups. Adjusted Cox and Poisson models included age, sex, dialysis vintage, and dialysis access. Mortality follow-up was calculated from the first available PTH measurement to death or the end of follow-up. Poisson models used follow-up time as an offset and robust standard errors. Arithmetic mean PTH was calculated as the unweighted mean of all available serial PTH measurements for each patient.

Supplementary Table S2. Missingness of functional assessments according to time-weighted mean PTH categories

Functional measure	<150 (n=33)	150-300 (n=64)	300-600 (n=64)	>600 (n=27)	p-value
Gait stability	9 (27.3%)	12 (18.8%)	5 (7.8%)	2 (7.4%)	0.039
Mobility aid use	9 (27.3%)	12 (18.8%)	5 (7.8%)	2 (7.4%)	0.039
Dressing	9 (27.3%)	14 (21.9%)	5 (7.8%)	2 (7.4%)	0.022
Bathing	9 (27.3%)	14 (21.9%)	5 (7.8%)	2 (7.4%)	0.022
Toileting	8 (24.2%)	14 (21.9%)	5 (7.8%)	2 (7.4%)	0.041
Overall mobility	8 (24.2%)	12 (18.8%)	5 (7.8%)	2 (7.4%)	0.077
Continence	7 (21.2%)	11 (17.2%)	5 (7.8%)	2 (7.4%)	0.163
Falls history	12 (36.4%)	17 (26.6%)	7 (10.9%)	3 (11.1%)	0.010
Norton score	8 (24.2%)	9 (14.1%)	4 (6.2%)	2 (7.4%)	0.076

Abbreviations: PTH, parathyroid hormone; TW, time-weighted. Values are presented as number and percentage of patients with missing data, n (%), within each time-weighted mean PTH category (<150, 150-300, 300-600, and >600 pg/mL). Missingness reflects the absence of documented functional assessment in the routine clinical record. P-values compare the proportion of missing observations across PTH categories using the Fisher-Freeman-Halton exact test. Missing values were not imputed.
